# Methicillin-resistant *Staphylococcus aureus* colonization of infectious and non-infectious skin and soft tissue lesions in patients in Tehran

**DOI:** 10.1186/s12866-021-02340-w

**Published:** 2021-10-18

**Authors:** Haniyeh Khalili, Shahin Najar-Peerayeh, Mona Mahrooghi, Parvin Mansouri, Bita Bakhshi

**Affiliations:** 1 Department of Bacteriology, Faculty of Medical Sciences, Tarbiat Modares University, Tehran, Iran; 2 Department of research, Skin and Stem Cell Research Center, Tehran University of Medical Sciences, Tehran, Iran

**Keywords:** HA-MRSA, CA-MRSA, Skin and soft tissue, Biofilm

## Abstract

**Background:**

The most common clinical manifestations of *Staphylococcus aureus* strains in the community are skin and soft-tissue infections. *S. aureus* could colonize the body sites and complicate the pathogenesis of skin diseases. *S. aureus* colonization is a risk factor for severe conditions such as bone and joint infections, pneumonia, bacteremia, and endocarditis. This study aimed to investigate the prevalence of *S. aureus* strains in skin and soft tissue infections and other skin disorders in patients referring to dermatology clinics and to evaluate the antibiotic resistance pattern and molecular characteristics of *S. aureus* isolates.

**Methods:**

Skin swabs were collected from the lesional sites in 234 outpatients referring to dermatology clinics in three hospitals in Tehran. Antibiotic susceptibility, biofilm formation, and hemolysis tests were performed for isolates. PCR was done for SCC*mec* typing, *agr* grouping, and virulence genes detecting.

**Results:**

The prevalence of *S. aureus* strains among patients with skin and soft-tissue infections and other skin lesions was 44.77% (30/67) and 44.91% (75/167), respectively. Also, 59 (56.19%) isolates were MRSA, 35.57% were HA-MRSA, and 30.5% were CA-MRSA. The *psmα* gene was more prevalent (62.8%) among isolates, followed by *hla*α (56.1%), *tsst-*1 (15.2%) *eta* (13.3%), *etb* (6.6%), and *pvl* (2.8%). The *agr* specificity groups I, II, III, and IV were identified in 49.5, 21.9, 11.4, and 14.2% of *S. aureus* isolates, respectively. Most (56%) *S. aureus* isolates produced a moderate biofilm, and 23.8% of them produced strong biofilms. α-hemolysin (46.6%), β-hemolysin (25.7%), γ-hemolysin (19%), and both α and β-hemolysin (5.7%) were also produced by isolates.

**Conclusion:**

The present study results indicated high colonization of skin lesions by HA-MRSA and CA-MRSA clones; MRSA strains were more resistant to antibiotics, contained various toxin genes, and were able to form biofilms. Therefore, they could play a vital role in the pathogenesis of various skin diseases; also, they could spread and cause infections in other body sites. Eradication and decolonization strategies could prevent recurrent infections and the spread of resistant strains and improve skin conditions.

## Introduction

*Staphylococcus aureus* is an opportunist bacterium that causes a variety of clinical infections, ranging from skin and soft-tissue and device-related infections to bacteremia and endocarditis [1, 2]. On the other hand, *S. aureus* is a member of the commensal bacteria of the mucosal microbiome. Asymptomatic colonization of anterior nares by *S. aureus* in healthy individuals is approximately 25–30%, while the colonization of other body sites is less frequent [3, 4]. When the skin microbiome is imbalanced, and pro-inflammatory cytokines change the skin environment, disorders such as atopic dermatitis, rosacea, and psoriasis appear. *S. aureus* is one of the most common bacteria found in these conditions, especially in atopic dermatitis [5].

*S. aureus* expresses several virulence factors that contribute to the colonization of the skin and invasion of epidermal barriers, including polysaccharide intercellular adhesin (PIA) molecular polymer, which triggers adhesion and biofilm formation. Also, *S. aureus* toxins, such as phenol-soluble modulins, exfoliative toxin A and B (EtA, EtB), toxic shock syndrome toxin 1 (TSST-1), hemolysins, and Panton-Valentine leukocidin (PVL) toxin, induce immune cell death and cytokine release [5, 6]. Additionally, methicillin-resistant *S. aureus* (MRSA) is a significant cause of skin and soft-tissue colonization and infections, especially community-associated MRSA (CA-MRSA), which is more virulent and grows faster than hospital associated-MRSA (HA-MRSA) [7, 8]. However, the actual role of virulence factors in the pathogenesis of different *S. aureus* clinical manifestations is unclear. A few studies have been conducted on *S. aureus* prevalence in skin and soft-tissue lesions in our country. Therefore, this study aimed to evaluate the prevalence of *S. aureus* in infectious and non-infectious skin and soft-tissue lesions in patients referring to dermatology clinics in Tehran and to evaluate antibiotic resistance pattern and molecular characteristics of *S. aureus* isolates.

## Results

### Characteristics of patients and bacterial isolates

In this study, 234 patients with skin disorders were categorized into two distinct groups based on their clinical impressions, including 67 patients with skin and soft-tissue infections (SSTIs) such as blister, impetigo, cellulitis, erysipelas, abscess, furuncles, necrotizing fasciitis, wounds, and insect/animal bites and 167 patients with non-infectious skin lesions such as eczema, erythematous skin lesions, psoriasis, drug reactions, erythema nodosum, insect/animal bites, and carcinoma. Table 1 shows the general characteristics of the patients included in this study. A total of 105 *S. aureus* isolates were collected from the patients: 30 (28.57%) isolates from SSTIs and 75 (71.42%) isolates from non-infectious skin lesions. The mean age of the patients with *S. aureus* colonization of skin lesions was 22.3 years (ranging from 30 days to 85 years). Also, 51.4% (54/105) of the patients were female (Table 1).

**Table 1:** Demographic characteristics of the two patient groups

Characteristics	Infectious Group	Non-infectious Group	Total	*P* ≤ .05, OR; 95% CI
Total patients	67(28.63%)	167(71.36%)	234(100%)	–
Female /Male	46/21	73/94	119/115	.001,1.571(1.240–1.989)
Median age years	23.5	21.7	22.3	–
Infant (≤2)	11(16.4%)	44(26.3%)	55(23.5%)	.105,0.623(0.342–1.132)
Children (3–18)	22(32.8%)	54(32.3%)	76(32.4%)	.941,1.015(0.676–1.525)
Adults (18<)	34(50.7%)	69(41.3%)	103(44%)	.189,1.228(0.912–1.653)
Patients with *S. aureus*	30(44.7%)	75(44.9%)	105(44.8%)	.985,0.997(0728–1.366)
Female /Male	20/10	34/41	54/51	.048,1.471(1.031–2.097)
Median age years	24.2	22.6	23.4	–
Infant (≤2)	6(20%)	20(26.6%)	26(24.7%)	.475,0.750(0.334–1.683)
Children (3–18)	9(30%)	20(26.6%)	29(37.6%)	.730,1.125(0.580–2.183)
Adults (18<)	15(50%)	35(46.6%)	50(47.6%)	.757,1.071(0.696–1.650)

The prevalence of *S. aureus* strains was 44.77% (30/67) and 44.91% (75/167) among patients with SSTIs and other skin lesions, respectively. There was no significant difference in *S. aureus* colonization of skin lesions between the two groups of patients (*p* = .985, OR = 0.997, 95% CI: 0728–1.366).

### Antibiotic susceptibility testing

**Fig. 1:**
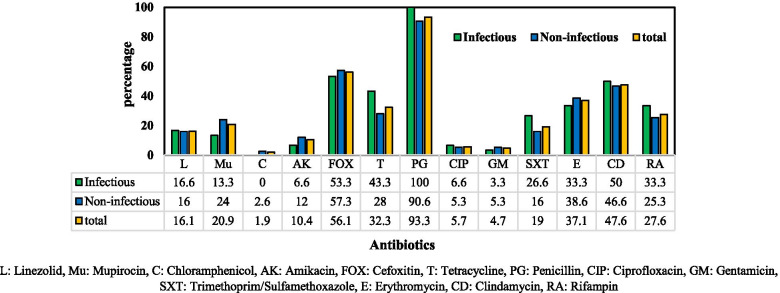
Antibiotic resistance pattern of *S. aureus* strains isolated from infectious and non-infectious lesions. L: Linezolid, Mu: Mupirocin, C: Chloramphenicol, AK: Amikacin, FOX: Cefoxitin, T: Tetracycline, PG: Penicillin, CIP: Ciprofloxacin, GM: Gentamicin, SXT: Trimethoprim/Sulfamethoxazole, E: Erythromycin, CD: Clindamycin, RA: Rifampin

Among 105 *S. aureus* isolates tested, the highest antibiotic resistance rate was related to penicillin (98, 93.3%), followed by cefoxitin (59, 56.1%), clindamycin (48, 47.6%), erythromycin (42, 37.1%), and tetracycline (34, 32.3%). Additionally, resistance to rifampin, mupirocin, and linezolid was observed in 26 (27.6%), 22 (20.9%), and 17 (16.1%) isolates, respectively. Fortunately, the isolates susceptibility to chloramphenicol (103, 98.1%), gentamicin (100, 95.3%), ciprofloxacin (99, 94.3%), and amikacin (94, 89.6%) was high (Fig.1). All the strains (100%) isolated from SSTIs were resistant to penicillin (Fig.1).

Among 105 isolates, 59 (56.1%) isolates were methicillin-resistant *S. aureus* (MRSA). The prevalence of MRSA among *S. aureus* strains isolated from SSTIs and other skin lesions was 53.3% (16/30) and 57.3% (43/75), respectively.

Penicillin resistance rate (93%) was similar in MRSA and MSSA strains (Table 2). However, MRSA strains were more resistant to the antibiotics tested compared to MSSA isolates, including amikacin (16% vs. 2%), trimethoprim/sulfamethoxazole (28% vs. 6%), clindamycin (62% vs. 28%), and rifampin (35% vs. 17%). High resistance to clindamycin (62%), rifampin (35%), and linezolid was observed in MRSA strain isolated from non-infectious skin lesions (Table 2). There was no significant difference regarding the prevalence of MRSA in skin lesions between the two groups of patients (*p* = .766, OR = 1.105, 95% CI: 0.571–2.139).

**Table 2:** Prevalence of antibiotics resistance among MRSA and MSSA strains from the two groups of lesions

MRSA					MSSA			
	Infectious Group ***N*** = 16	Non-infectious Group ***N*** = 43	χ^**2**^, ***P***-Value	Total ***N*** = 59	Infectious Group ***N*** = 14	Non-infectious Group ***N*** = 32	χ^**2**^, ***P***- Value	Total ***N*** = 46
L	3(18.7%)	8(32%)	.990	11(18.6%)	2(14.2%)	4(12.5%)	.869	6(13.3%)
Mu	1(6.2%)	12(27.9%)	.074	13(22%)	3(21.4%)	6(18.7%)	.833	9(19.5%)
C	0	2(4.6%)	.278	2(3.3%)	0	0	–	0
AK	2(12.5%)	8(18.6%)	.578	10(16.9%)	0	1(3.1%)	.504	1(2.1%)
T	8(50%)	13(30.2%)	.159	21(35.5%)	5(35.7%)	8(25%)	.458	13(28.2%)
PG	16(100%)	39(90.6%)	.206	55(93.2%)	14(100%)	29(90.6%)	.236	43(93.4%)
CIP	2(12.5%)	3(6.9%)	.498	5(8.7%)	0	1(3.1%)	.504	1(2.1%)
GM	1(6.2%)	4(9.3%)	.708	5(8.7%)	0	0	–	0
SXT	7(43.7%)	10(23.2%)	.122	17(28.8%)	1(7.1%)	2(6.2%)	.910	3(6.5%)
E	8(50%)	16(37.2%)	.374	24(40.6%)	5(35.7%)	13(40.6%)	.754	18(39.1%)
CD	11(68.7%)	28(65.5%)	.793	37(62.7%)	2(14.2%)	9(28.1%)	.311	11(23.9%)
RA	6(37.5%)	15(34.8%)	.852	21(35.5%)	1(7.1%)	4(12.5%)	.591	5(10.8%)

*L* linezolid, *Mu* mupirocin, *C* chloramphenicol, *AK* amikacin, *FOX* cefoxitin, *T* tetracycline, *PG* penicillin, *CIP* ciprofloxacin, *GM* gentamicin, *SXT* trimethoprim/sulfamethoxazole, *E* erythromycin, *CD* clindamycin, *RA* rifampin

Most MRSA strains were simultaneously resistant to more than three antimicrobial classes. One MRSA isolate was resistant to all the antibiotics tested, except amikacin. Also, six MRSA isolates were simultaneously resistant to linezolid, mupirocin, penicillin, clindamycin, and rifampin. Additionally, two MSSA isolates were susceptible to all the antibiotics tested, and two other MSSA isolates were susceptible to all the antibiotics tested, except erythromycin.

### Detection of resistance genes

MRSA strains isolated from SSTIs belonged to SCC*mec* II (4, 25%), SCC*mec* III (3, 18.7%), SCC*mec* IVa (1, 6.2%), and SCC*mec* IVb (3, 18.7%), and 5 (31.2%) isolates were untypeable. Also, SCC*mec* I (2, 4.6%), SCC*mec* II (4, 9.3%), SCC*mec* III (8, 18.6%), SCC*mec* IVa (4, 9.3%), SCC*mec* IVb (4, 9.3%), SCC*mec* IVc (1, 2.3%), SCC*mec* IVd (2, 4.6%), and SCC*mec* V (3, 6.9%) as well as 11 (25.5%) unknown isolates were identified in the strains isolated from non-infectious skin lesions.

The *int*1gene was identified in 42 (40%) isolates, among which 29 isolates were MRSA, and 13 isolates were MSSA.

### *Agr* grouping and detection of virulence genes

The toxin genes detected in the isolates are shown in Table 3. The most prevalent gene among 105 isolates was *psmα* (66, 62.8%), followed by *hlaα* (59, 56.1%), *tsst-1*(16, 15.2%), *eta* (14, 13.3%), *etb* (7, 6.6%), and *pvl* (3, 2.8%). The *pvl* gene was detected only in MSSA isolates, and exfoliative toxin genes were more prevalent in MSSA isolates (Table 3). The prevalence of *hlaα* gene was significantly higher in the strains from non-infectious skin lesions than in the strains from infectious skin lesions (*p* = 0.034; χ^2^ = 0.397; 95% CI: 0.167–0.946). There was no significant difference regarding the prevalence of other virulence genes between the two groups of isolates (Table 3).

**Table 3:** Prevalence of toxin genes, *agr* groups, and biofilm formation in *S. aureus* isolates from the two groups

	Infectious Group *N* = 30	Non-infectious Group *N* = 75	χ^2^ *P*-Value	MRSA N = 59	MSSA N = 46	χ^2^ *P*-Value	Total *N* = 105
tsst-1	4(13.3%)	12(16%)	.732	13(23.2%)	3(6.5%)	.028	16(15.2%)
pvl	2(6.6%)	1(1.33)	.138	0	3(6.5%)	.047	3(2.8%)
Eta	3(10%)	11(14.6%)	.525	7(11.8%)	7(15.2%)	.616	14(13.3%)
etb	3(10%)	5(6.6%)	.561	2(3.3%)	5(10.8%)	.127	7(6.6%)
hlaα	12(40%)	47(62.6%)	.034	38(64.4%)	21(45.6%)	.055	59(56.1%)
psmα	20(66.6%)	46(61.3%)	.609	38(64.4%)	28(60.8%)	.710	66(62.8%)
agr *I*	11(36.6%)	41(54.6%)	.096	25(42.3%)	27(58.6%)	.097	52(49.5%)
agr *II*	9(30%)	14(18.6%)	.205	10(16.9%)	13(28.2%)	.164	23(21.9%)
agr *III*	4(13.3%)	8(10.6%)	.698	11(18.6%)	1(2.1%)	.008	12(11.4%)
agr *IV*	3(10%)	12 (16%)	.427	10(16.9%)	5(10.8%)	.377	15(14.2%)
*S-biofilm*	6(20%)	19(25.3%)	.562	19(32.2%)	6(13%)	.022	25(23.8%)
*M-biofilm*	15(50%)	44(58.6%)	.419	24(40.6%)	35(76%)	.000	59(56.1%)
*W-biofilm*	6(20%)	9(12%)	.290	13(22%)	2(4.3%)	.010	15(14.2%)
*No-biofilm*	3(10%)	3(4%)	.231	3(5%)	3(6.5%)	.757	6(5.7%)

*S* strong, *M* moderate, *W* weak

The *agr* specificity groups I, II, III, and IV were identified in 49.5, 21.9, 11.4, and 14.2% of the isolates, respectively, and 3 (2.8%) isolates were untypeable (Table 3).

### Biofilm formation and hemolysis pattern

Most (59/105, 56%) *S. aureus* isolates produced a moderate biofilm, and 23.8% (25/105) were strong-biofilm producers (Table 3). Strong biofilm-producing isolates were more prevalent among MRSA strains (19/59; 32%) than MSSA isolates (6/46, 13%).

The prevalence of strong biofilm-producing isolates in infectious and non-infectious skin lesions was 20% (6/30) and 25% (19/75), respectively. Among 25 strong biofilm-producing isolates, the *tsst-1* gene was found in 5 (20%) isolates, *hlaα* in 16 (64%) isolates, *eta* and *etb* in 6 (24%) isolates each, and *pvl* in 1 (4%) isolate. Additionally, 36% (9/25) of strong biofilm-producing isolates were CA-MRSA, 24% (6/25) were HA-MRSA, 20% (5/25) were untypeable MRSA, and 20% (5/25) were MSSA. Biofilm formation was significantly higher in MRSA strains (Table 3).

All the isolates also produced hemolysin, including α-hemolysin (49/105, 46.6%), β-hemolysin (27/105, 25.7%), γ-hemolysin (20/105, 19%), and both α and β-hemolysin (9/105, 5.7%).

## Discussion

In this study, the presence of *S. aureus* strains in various skin and soft tissue lesions was evaluated. *S. aureus* SSTIs have various clinical presentations such as blister, impetigo, cellulitis, abscess, furuncles, and necrotizing fasciitis. This bacterium is not a member of the skin and soft tissue normal microbiota; however, its colonization in the skin and soft tissues complicates many skin diseases pathogenesis or persistence [9]. In our country, a few studies have been conducted to evaluate the presence of *S. aureus* in skin and soft tissue lesions. In this study, *S. aureus* colonization was evaluated in two groups of patients with infectious and non-infectious lesions. The results showed that the rate of *S. aureus* colonization was almost the same in both groups of patients (44.7% vs. 44.9%). In other studies in Iran, the prevalence of *S. aureus* has been reported to be 22.6 and 51.3% in SSTIs in Shiraz and Tehran, 33% in atopic dermatitis, and 59.1% in pemphigus vulgaris legions, respectively [10–13], while the prevalence of MRSA has been reported to be 46.8 and 60% in SSTIs in Shiraz and Tehran, 42.2% in pemphigus vulgaris, and 33% in atopic dermatitis lesions [10–13]. Unexpectedly, the prevalence rate of MRSA strains was higher in non-infectious lesions (57.3%) than in infectious lesions (53.3%). This is likely due to underlying chronic diseases in patients in the non-infectious group, such as eczema, psoriasis, and chronic erythema nodosum.. In some studies in Geneva, Beijing, and Vancouver, the incidence rate of MRSA isolates has been reported to be 7, 3, and 54% in SSTIs, respectively [14–16]. The high prevalence rate of MRSA strains in outpatients in this study and other research in Iran could be extremely worrying due to the relatively high resistance of MRSA strains to the antibiotics tested (Table 2), especially to clindamycin (72%), and to some extent, to mupirocin (22%); treatment or decolonization efforts for MRSA strains seems to be problematic. Decolonization with chlorhexidine body washes or diluted sodium hypochlorite (bleach) baths is recommended [9].

Resistance to penicillin was very high, and all the strains isolated from infectious lesions were completely resistant to penicillin (Fig. 1). *S. aureus* strains isolated from non-infectious lesions were more resistant to the antibiotics tested, and 57% of them were MRSA. This group of patients had various chronic diseases, and most of them had a history of hospitalization. SCC*mec* type I to V were detected in MRSA strains isolated from infectious and non-infectious lesions, indicating that both CA-MRSA and HA-MRSA are circulating in Tehran community. Some studies have shown the simultaneous presence of CA-MRSA and HA-MRSA in the community and hospital settings [17–19]; therefore, the use of SCC*mec* type as a marker to differentiate between CA-MRSA and HA-MRSA may not be useful.

Regarding virulence genes, the *pvl* gene was detected only in three strains, all of which were MSSA; two strains were isolated from four- and six-month-old newborns, and one strain was isolated from eczema lesions of a 65-year-old man. PVL has been reported to be associated with MRSA strains, especially CA-MRSA [15]. However, recent research has shown that PVL is found in both methicillin-sensitive and -resistant strains in the community and healthcare settings, and its incidence rate depends on the sample size and the geographical regions [20–23]. Therefore, the PVL-bacteriophage acquisition event is probably independent of the *mec*A gene acquisition.

The presence of toxin genes, such as *psmα, hla*α, *tsst-*1, *eta,* and *etb,* was also evaluated in this study. Bacterial toxins may impair the integrity of the skin’s defense barriers, allowing bacteria and antigens to penetrate in the sub-epithelium. Also, the toxic shock syndrome toxin-1 (TSST-1) is a strong super-antigen that induces an inflammatory response by activating the immune system [24–26]. Generally, 15% of *S. aureus* isolates carried the *tsst-*1 gene; also, the *psmα* and *hla*α genes were present in more than 55% of the isolates. Exfoliative toxin genes were identified in 20% of the isolates, and α, β, and γ cytotoxins were produced by the isolates. All of these cytotoxins are likely to play a role in the structural and immune condition of patients’ skin.

Biofilm formation is associated with many *S. aureus* infections. Biofilm formation protects microorganisms against natural skin antimicrobials, immune responses, environmental stresses, and antibiotic treatment [27–29]. In general, 80% of the isolates in this study produced strong or moderate biofilm, and no statistical differences were found in biofilm formation between the strains isolated from the two groups of patients. However, biofilm formation was significantly higher in MRSA strains than in MSSA (Table 3). Biofilm formation is a typical feature of *S. aureus* isolates from various clinical specimens [27–30]. After the maturation of biofilm, *S. aureus* isolates could disseminate and colonize new regions. Therefore, biofilm formation is necessary for colonization and infection.

Evaluation of *agr* specificity groups detected in this study showed that the frequency of *agr* specificity group I (49.5%) was higher than other *agr* groups among the isolates. *Agr* group I is dominant in most *S. aureus* isolates from various clinical specimens [31, 32]. The prevalence of *agr* specificity group III was significantly (*p* = .008) higher in MRSA strains than in MSSA; further studies are needed to confirm the association between MRSA strains and *agr* specificity group III in skin and soft tissue lesions.

Although in this study, the relationship between *S. aureus* isolates and the type of skin lesions was not evaluated by genotypic analysis such as MLST, PFGE, or other typing methods, but high clonal diversity has been reported by researchers among *S. aureus* isolates from various skin and soft tissue lesions. For example, Zhao et al. (2012) [15] suggested similar epidemiology for community-acquired and hospital-acquired *S. aureus* infections. Yeung et al. (2011) [33] concluded there was no predominant clonal type among *S. aureus* isolates from atopic dermatitis lesions. However, SCC*mec* typing, *agr* grouping, and virulence typing results in the present study indicated no significant difference between the isolates from various skin lesions, except for *hla*α gene that was significantly more prevalent in non-infection legions. Further studies are needed to investigate the relationship between isolates and different skin legions in our country.

## Conclusion

In this study, the prevalence and molecular characteristics of MSSA and MRSA strains isolated from infectious and non-infectious skin and soft-lesions were investigated in Iranian dermatology clinics. The results indicated high colonization of skin lesions by HA-MRSA and CA-MRSA clones; MRSA strains were more resistant to antibiotics commonly used in the treatment of *S.aureus* infection. Furthermore, these isolates contained various toxin genes and were able to form biofilm, which are considered as essential factors for generating antibiotic-resistant infections. *S. aureus* could play a major role in the pathogenesis of various skin diseases; also, it could spread and cause infections in other body sites. The present study results showed a high prevalence rate of MRSA in outpatients, given that in the past, there was an emphasis on recognizing these isolates in skin and soft-tissue lesions and determining their antibiogram profile. Eradication and decolonization strategies could prevent recurrent infections and the spread of resistant strains and improve skin conditions.

## Material &Methods

### Characteristics of patient and bacterial isolates

Skin swabs were collected from the lesional sites in outpatients by dermatologists in three hospitals (two general and one pediatric hospitals) in Tehran during April 2016–2017. Demographic and clinical data of patients were recorded. Patients who took antibiotics 2 weeks prior to sampling were excluded. The collected swab samples were transported promptly in thioglycolate broth to the clinical microbiology laboratory. After overnight incubation at 37 °C, the samples were cultured on blood agar medium at 37 °C. *S. aureus* strains was identified using the following tests, including gram staining, catalase test, mannitol fermentation, slide and tube coagulase tests, DNase production, and presence of the *nuc* gene [34].

### Antibiotic susceptibility testing

Antimicrobial susceptibility test was performed for the isolates according to the Clinical and Laboratory Standards Institute [35]. Antibiotic disks used were as follows; clindamycin (2 μg), mupirocin (200 μg), chloramphenicol (30 μg), amikacin (30 μg), penicillin (10 μg), gentamicin (10 μg), tetracycline (30 μg), ciprofloxacin (5 μg), trimethoprim-sulfamethoxazole (1.25/23.75 μg), cefoxitin (30 μg), rifampin (5 μg), erythromycin (15 μg), and linezolid (30 μg) (MAST UK). *S. aureus* ATCC25923 was used as a quality control.

### Detection of resistance genes

The *mec*A and class I integron genes were detected among the isolates by PCR. MRSA strains were typed for SCC*mec.* The specific primers and thermal profiles employed for PCR detection of these genes are shown in Table 4.

**Table 4:** Primers used in this study

Primer	Sequence (5′ → 3′)	Size (bp)	Ref.
*mecA*	F: GTGAAGATATACCAAGTGATT R: ATGCGCTATAGATTGAAAGGA	146	[36]
SCC*mec* I	F: GCTTTAAAGAGTGTCGTTACAGG R: GTTCTCTCATAGTATGACGTCC	613	
SCC*mec* II	F: CGTTGAAGATGATGAAGCG R: CGAAATCAATGGTTAATGGACC	398	
SCC*mec* III	F: CCATATTGTGTACGATGCG R: CCTTAGTTGTCGTAACAGATCG	280	
SCC*mec* Iva	F: GCCTTATTCGAAGAAACCG R: CTACTCTTCTGAAAAGCGTCG	776	
SCC*mec* IVb	F: TCTGGAATTACTTCAGCTGC R: AAACAATATTGCTCTCCCTC	493	
SCC*mec* IVc	F: ACAATATTTGTATTATCGGAGAGC R: TTGGTATGAGGTATTGCTGG	200	
SCC*mec* IVd	F: CTCAAAATACGGACCCCAATACA R: TGCTCCAGTAATTGCTAAAG	881	
SCC*mec* V	F: GAACATTGTTACTTAAATGAGCG R: TGAAAGTTGTACCCTTGACACC	325	
panF	F: ATGCACATGGTGCACATGC	–	[37]
*agr* I-R	R: GTCACAAGTACTATAAGCTGCGAT	440	
*agr* II-R	R: GTATTACTAATTGAAAAGTGCCATAGC	572	
*agr* III-R	R: CTGTTGAAAAAGTCAACTAAAAGCTC	406	
*agr* IV-R	R: CGATAATGCCGTAATAC CCG	588	
*tsst-1*	F: TTATCGTAAGCCCTTTGTTG R: TAAAGGTAGTTCTATTGGAGTAGG	398	[38]
*hla-α*	F: CGGTACTACAGATATTGGAAGC R: TGGTAATCATCACGAACTCG	744	[38]
*psm-a*	F: TATCAAAAGCTTAATCGAACAATTC R: CCCCTTCAAATAAGATGTTCATATC	176	[39]
etA	F: CTAGTG CATTTGTTATTCAAGACG R: TGCATTGACACCATAGTACTTATTC	119	[40]
*etB*	F: ACGGCTATATACATTCAATTAATG R: AAAGTTATTCATTTAATGCACTGTCTC	200	[40]
*Int1*	F: CCTCCCGCACGATGATC R: TCCACGCATCGTCAGGC	188	[41]

### *Agr* grouping and detection of virulence genes

The presence of *pvl*, *eta*, *etb, tsst-1, hla-α*, and *psmα* genes was detected among the isolates by polymerase chain reaction (PCR). *agr* grouping was performed for all the isolates. The specific primers and thermal profiles employed for PCR detection of these genes are shown in Table 4.

### Biofilm formation and hemolysis pattern

The biofilm formation ability of all the isolates was evaluated using the microtiter plate method [34]. To do so, the tryptic soy broth (TSB) supplemented with 1% (w/v) glucose containing 107 CFU/mL of each isolate was distributed into 96-well polystyrene microtiter plates and incubated at 37 °C for 24 h while shaking at 120 rpm. After twice washing and then air-drying, a 0.1% crystal violet solution was added and after 45 min washed three times, and then the ethanol-acetone solution was added. A microplate reader measured the absorbance of each well at OD570 nm after 45 min. *S. aureus* ATCC 35556 and *S. epidermidis* ATCC 12228 were used as a positive and negative control, respectively. The tests were done in duplicate at two independent times.

*S. aureus* hemolysis patterns were characterized in the blood agar medium containing 5% washed rabbit red blood cells. All tests were done at two independent times.

### Statistical analysis

SPSS software Version 23 (SPSS Inc., Chicago, IL, USA) was used for statistical analysis. Differences in proportions were evaluated by employing the Chi-square test. Univariate analysis was performed by logistic regression. A *p*-value < .05 was considered statistically significant.

## Data Availability

The authors confirm that the data supporting the findings of this study are available within the article.

## Abbreviations

*S. aureus*: *Staphylococcus aureus*
EtA: Exfoliative toxin A
EtB: Exfoliative toxin B
TSST-1: Toxic shock syndrome toxin 1
PVL: Panton valentine toxin
MRSA: Methicillin-resistant *Staphylococcus aureus*
HA-MRSA: Health care associated or hospital- associated MRSA
CA-MRSA: Community-associated MRSA
SCC*mec*: Staphylococcal cassette chromosome *mec*
